# The Sex-Specific Splicing of Doublesex in Brine Shrimp *Artemia franciscana*

**DOI:** 10.3390/genes13111997

**Published:** 2022-11-01

**Authors:** Dung Nguyen Viet, Olivier Christiaens, Stephanie De Vos, Guy Smagghe, Peter Bossier

**Affiliations:** 1 Research Institute for Aquaculture No. 2, 116 Nguyen Dinh Chieu Street, Dakao Ward, District 1, Ho Chi Minh City 71007, Vietnam; 2 Laboratory of Aquaculture & Artemia Reference Center, Ghent University, Coupure Links 653, 9000 Ghent, Belgium; 3 Laboratory of Agrozoology, Department of Plants and Crops, Faculty of Bioscience Engineering, Ghent University, Coupure Links 653, 9000 Ghent, Belgium

**Keywords:** *Artemia franciscana*, *Doublesex*, sex-specific splicing

## Abstract

The understanding of sex determination and differentiation in animals has recently made remarkable strides through the use of advanced research tools. At the gene level, the Mab-3-related transcription factor (*Dmrt*) gene family, which encodes for the typical DNA-binding doublesex/Mab-3 (DM) domain in their protein, is known for its contribution to sex determination and differentiation in insects. In this study, DNA-binding DM domain screening has identified eight transcripts from *Artemia franciscana* transcriptomic that encode proteins containing one conserved DNA-binding DM domain. The genome mapping confirmed that these eight transcripts are transcribed from six different loci on the *A. franciscana* genome assembly. One of those loci, the Af.dsx-4 locus, is closely related to *Doublesex*, a gene belonging to the *Dmrt* gene family. This locus could be transcribed into three alternative transcripts, namely Af.dsx^4^, Af.dsx^F^ and Af.dsx^M^. While Af.dsx^4^ and Af.dsx^F^ could putatively be translated to form an identical Af.dsx^F^ protein of 186 aa long, Af.dsx^M^ translates for an Af.dsx^M^ protein of 289 aa long but shares a DNA-binding DM domain. Interestingly, Af.dsx^F^ and Af.dsx^M^ are confirmed as sex-specific transcripts, Af.dsx^F^ is only present in females, and Af.dsx^M^ is only present in male individuals. The results suggest that the sex-specific splicing mechanism of the *doublesex* described in insects is also present in *A. franciscana*. Af.dxs-4 locus can be used in further studies to clarify the sex determination pathways in *A. fracnciscana*.

## 1. Introduction

The Artemia genus contains species with sexual as well as parthenogenetic reproduction modes. Artemia has a short life cycle, and is cultured easily under laboratory conditions. This species is being used as an animal model to discover genes involved in biological processes. *Artemia* has a WZ-ZZ sex-determining system which means that males are homogametic (ZZ), while females are heterogametic (ZW) [1,2] such as other crustacean species including *Cherax quadricarinatus* [3], *Penaeus monodon* [4], *Macrobrachium rosenbergii* [5] and *Eriocheir sinensis* [6]. In an effort to explore the molecular mechanism of sex determination in *Artemia*, a few molecular biology techniques have been employed to understand this mechanism. The Z-chromosome of *Artemia. franciscana* has recently been characterized by detecting genomic regions that show lower genomic coverage in female than in male samples, and regions that harbor an excess of female-specific SNPs [7]. Eight sex-linked AFLP marker alleles have been reported in *A. franciscana* that are inherited from the female parent [1]. Some genes related to sexual differentiation in *A. franciscana* have been identified and shown as sex-biased gene expression [8]. Masculinizer gene (*Ar-Masc)* was identified in *A. franciscana* [9], the MASC protein encoded in the Z chromosome activates a sex-determining gene that regulates gene splicing of *Doublesex* (*dsx*) into the male-specific isoform in the silkworm *Bombyx mori* [10]. Until now, the sex-determining genes in *A. franciscana* have not yet been identified and no genetic sex-specific marker is reported.

Doublesex and Mab-3-related transcription factor (*Dmrt*) genes family are reported to be very highly conserved, as well as essential for sexual development in mammal, fish, and insects. In Dmrt genes family, *Dmrt1*, *Dmrt7*, and *Dmrt8* are only found in vertebrate; *dmrt2a/2b*, *dmrt3*, *dmrt4/5* and *dmrt93B* are commonly present in invertebrate bilateria; whereas *Dmrt1* and *dsx* have suggested independent evolution for sex determination and differentiation in the Dmrt gene family [11]. *Doublesex* bearing a doublesex/Mab-3 (DM) domain, regulates the sexual differentiation in *Drosophila melanogaster*, was the first Dmrt family member to be discovered [12]. Dsx and another two Dmrt genes including *Dmrt99B* and *Dmrt11E* could only be found in arthropod. Dsx gene is a critical transcription factor considered to be at the end of the sex determination cascade in many other insect species as a master gene in genotypic sex determination pathway. Dmrt genes family are involved in the formation of sex-linked traits including sexual behavior, gonadal development and sex-specific morphology in many insects [13]. In *D. melanogaster*, dsx gene is transcribed to produce a common primary transcript that is alternatively spliced and polyadenylated to yield male (*dsxM*) and female (*dsxF*) specific transcript [12]. *Dsx* and *Dmrt* have been characterized in a few crustacean species including *M. rosenbergii* [14,15,16,17], *M. nipponense* [18], *E. sinensis* [19], *F. chinensis* [20], *Litopenaeus. vannamei* [21], and four *Daphnia* species [22]. In *F. chinensis*, *Dsx* exhibited a sex-biased expression pattern in different tissues and its expression level increased along with developmental stages. Its binding site was identified on the promoter region of insulin-like androgenic gland gene [20]. Two dsx genes have been identified in *D. magna* (*DapmaDsx1* and *DapmaDsx2*), which are expressed in males but not in females. *DapmaDsx1* has been reported as a sex determination gene which is responsible for the male trait development [22]. Recently, *Sagmariasus verreauxi*, a heterogametic sex-linked *iDMY/iDmrt1* has been recently reported, in which the dominant negative suppression of *iDMY* over its autosomal *iDmrt1* paralogue might play an important key in the mechanism of determines sex this species [23]. These findings suggest the Doublesex and Mab-3-related transcription factor genes is good clues to look for the sex-determining genes in Artemia. In this study, the Dsx/Dmrt gene family is a target candidate for studying the sex determination mechanism in *A. franciscana*. We are looking for sex-specific splicing of Dsx/Dmrt gene family in the transcriptome database of *A. franciscana*. The expression profile of those genes was evaluated in both males and females using RT-PCR.

## 2. Materials and Methods

### 2.1. Animal Culture

*A. franciscana* cysts (Great Salt Lake, UT, USA) were hydrated in autoclaved seawater with strong aeration at 28 °C until hatching (approximately 20–24 h). The nauplii (larvae) were then transferred to a 5 L plastic tank with autoclaved seawater for rearing. The larvae were cultured at 28 °C in autoclaved seawater with aeration under fluorescent light and were fed with live marine microalgae *Tetraselmis suecica*. At 15–20 DAH (days after hatching), the juvenile females (before egg-sac formation) were identified by their brown-red color. At the juvenile stage, 20 individuals (10 males and 10 females) were analyzed separately to confirm sex-specific transcripts by RT-PCR. Adult males and females are morphologically completely different 20–25 DAH with the hooked grasper phenotype in males and the egg-sac in females. At the adult stage, pooled samples of the whole body of 10 adult females or 10 adult males were used to confirm sex-specific transcripts by RT-PCR.

### 2.2. Total RNA Extraction and cDNA Synthesis

The total RNA of the samples (the hydrated cysts, nauplii, juveniles and adults) was prepared using the RNeasy mini kit (Qiagen, Hilden, Germany). The total RNA was used to synthesize cDNA in a 20 μL reaction by using poly-T primer and RevertAid H Minus First Strand cDNA Synthesis Kit (Thermal Scientific, Waltham, MA, USA). The 20 μL of reaction mixture contained 4 μL of 5X reaction buffer, 2 μL of 10 μM dNTPs mix, 20 units of ribonuclease inhibitor, 200 units of RevertAid H Minus M-MuLV Reverse Transcriptase, 1 μL of oligo-dT primer and 500 ng of total RNA. Subsequently, the reaction mixture was incubated for 70 min at 42 °C. The reaction was terminated by heating at 70 °C for 5 min and then cooled to 4 °C. Complementary deoxyribonucleic acids (cDNA) were then used as a template in PCR for further steps.

### 2.3. Identification of Putative of Dsx/Dmrt Transcripts

The primary amino acid sequences of *doublesex* from *Daphnia magna* were used for BLAST against the *A. franciscana* transcriptome database of the Laboratory of Aquaculture & Artemia Reference Center, Ghent University, Ghent, Belgium [24]. The identity of the selected hit contigs was subsequently verified using the BLASTX algorithm with default parameters on the nucleotide database of the National Center for Biotechnology Information (NCBI) [25]. Contigs matching Dsx/Dmrt gene family were selected for coding DNA sequence (CDS) analysis. The amino acid sequence of the selected contigs and open reading frame (ORF) was determined by the translation tool from the website Expasy (http://web.expasy.org/translate accessed on 26 January 2017). For contigs containing a full coding sequence (CDS), PCR primers were designed for isolation and sequencing. In contrast, for contigs containing only a partial CDS, their respective full-length cDNAs were identified by 3′ and 5′ rapid amplification of cDNA ends (RACE).

### 2.4. Isolation Full-Length of Dsx/Dmrt Transcript

Based on contigs containing only a partial CDS, gene-specific primers (Table 1) were designed to isolate the UTR (UnTranslated Region) using the SMARTer RACE Kit (Clontech, Palo Alto, CA, USA) with a modified protocol. The cDNA of each transcript from different lifecycle stages was used as a template for the PCR reaction to increase the chance of transcript amplification. Briefly, the 20 µL cDNA reaction for the isolation of the 5′UTR contained 4 µL 5X First-Strand Buffer, 0.5 µL DTT (100 mM), 1 µL dNTPs (20 mM) 5′-CDS Primer A, 10 μL total RNA, 1 µL of the SMARTer II A Oligonucleotide, 0.5 µL RNase Inhibitor (40 U/µL) and 2 µL SMARTScribe Reverse Transcriptase (100 U). The mixture was incubated at 42 °C for 70 min. The cDNA reaction for the isolation of the 3′UTR followed the same protocol as used for the isolation of the 5′UTR, except that SMARTer II A Oligonucleotide was not added, and the 5′-CDS Primer was replaced by 3′-CDS Primer from the kit. Subsequently, a PCR reaction (50 μL) was carried out to amplify the UTR sequence: 1 μL cDNA (for 5′ end UTR cDNA or 3′end UTR cDNA), 5 μL PCR buffer, 1 μL dNTPs (10 mM), 5 μL 10X UMP primer, 0.5 mM gene-specific primer (for 5′UTR or 3′UTR) and 0.25 μL Dream*Taq* DNA polymerase. The PCR thermal cycle was: denaturation for 3 min at 95 °C, followed by 35 cycles of amplification; 30 s at 95 °C; 30 s at 56 °C; and 2 min at 72 °C. The reaction was extended for 10 min at 72 °C and then cooled to 4 °C. The UTR PCR products were purified from agarose gel using the Wizard^®^ SV gel and PCR Clean-up System (Promega, Mandison, WI, USA) for subsequent, direct sequencing. The full length of the UTR sequence was assembled by Vector NTI software (Invitrogen, Waltham, MA, USA). For mapping analysis, the full length of the isolated transcript was mapped to the genome of *A. franciscana* (Unit of Polar Genomics, Korea Polar Research Institute, version 1.0, http://antagen.kopri.re.kr/project/genome_info_iframe.php?Code=AF01 accessed on 26 June 2022) using BLASTN with default parameters.

### 2.5. Phylogenetic Analysis

The phylogenetic tree was generated using website NGPhylogeny (https://ngphylogeny.fr, accessed on 17 September 2022), a phylogeny software based on the maximum-likelihook [26], wherein the usual bootstrapping procedure is replaced by a fast approximate likelihood ratio test (aLRT), which is proven to be a good alternative to the (time-consuming) bootstrap analysis. The putative polypeptide sequence from the isolated transcripts and known polypeptide sequences of homologs from NCBI were introduced under FASTA format.

### 2.6. Transcription Proof of Dsx/Dmrt Transcript

Gene-specific PCR primers was designed for each *Dsx/Dmrt* gene to amplify the complete transcript (Table 1). The applied PCR conditions were the same for all genes in this study. In addition, the PCR reaction contained 150 ng cDNA, 5 μL reaction buffer, 1 μL dNTPs (10 mM), 0.5 mM gene-specific primers, (Table 1) and 0.25 μL DreamTaq DNA polymerase (Thermal Scientific, Waltham, MA, USA). The PCR reaction was carried out in a thermal cycler (2720 Thermal Cycler (Thermal Scientific, Waltham, MA, USA) programmed for 94 °C for 4 min; 35 cycles at 94 °C for 30 s, 55 °C for 60 s, and 72 °C for 5 min; followed by 72 °C for 10 min. The amplicons were then separated on agarose gel and cleaned using Wizard^®^ SV Gel and PCR Clean-Up System (Promega, Madison, WI, USA). These cleaned amplicons were used directly for sequencing by the Sanger sequencing method using the same PCR primers.

## 3. Results

### 3.1. Identification of Dsx/Dmrt Transcripts

Six contigs encoding for polypeptides with a Doublesex/Mab-3 domain [27] were found in the *A. franciscana* transcriptome. These contigs were named doublesex-1, doublesex-2, doublesex-3, doublesex-4, doublesex-5 and doublesex-6. Based on the nucleotide sequence, only doublesex-3, doublesex-5 and doublesex-6 contained a full CDS encoding for the full putative polypeptide of the protein, while the others contained only a partial CDS. Therefore, the full doublesex-1, doublesex-2, and doublesex-4 cDNA sequences were then identified by the RACE method. Interestingly, RACE results revealed three different isoform from the doublesex-4 contig, namely doublesex-4α isoform A of 920 bp, doublesex-4α isoform B of 1010 bp; and doublesex-4β isoform C of 1156 bp. Their nucleotide sequences were submitted to NCBI GenBank as doublesex-1 (*dsx-1*) (MF287957), doublesex-2 (*dsx-2*) (MF287958), doublesex-3 (*dsx-3*) (MF287959), doublesex-4α isoform A (*dsx-4αA*) (MF287960), doublesex-4α isoform B (*dsx-4αB*) (MF993793), doublesex-4-β isoform C (*dsx-4βC*) (MF287961), doublesex-5 (*dsx-5*) (MF287962) and doublesex-6 (*dsx-6*) (MF287963).

**Figure 1:**
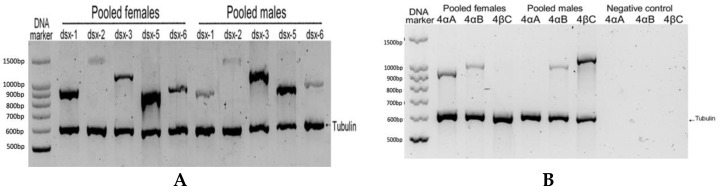
The evidence of transcription of *Dsx/Dmrt* genes from the cDNA samples of the pooled adult females and pooled adult males in *A. franciscana*. (**A**): The presence of *dsx*-1, *dsx*-2, *dsx*-3, *dsx*-5 and *dsx*-6 transcripts by semi-quantitative PCR (tubulin was used as the internal control). (**B**): The alternative transcript of *A. franciscana Af.dsx-4*, including *dsx-4αA*, *dsx-4αB* and *dsx-4βC,* confirmed the presence by PCR from the cDNA of the pooled adult animals.

The presence of the isolated Dsx/Dmrt transcripts were confirmed in adult *A. franciscana* animals by PCR method. The sex specific PCR analysis was conducted on cDNA from whole body RNA extraction of pooled male or female adults showed the presence of Dsx-1, Dsx-2, Dsx-3, Dsx-5 and Dsx-6 transcripts in both sexes (Figure 1A). However, dsx-4βC transcript could only be seen in the pooled males, while dsx-4αA transcript could only be found in the pooled females. Interestingly, dsx-4αB transcript was present in both sexes (Figure 1B).

### 3.2. Genome Mapping and Putative Protein Translation

**Figure 2:**
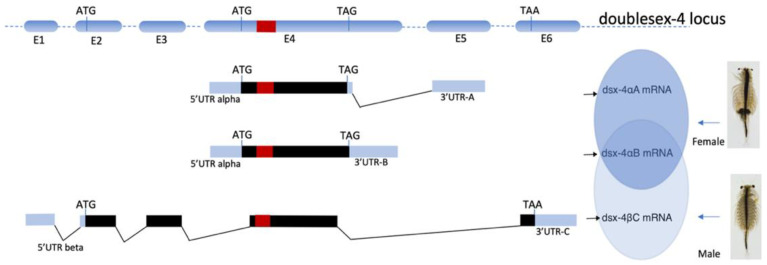
The alternative splicing model of the *Af.dsx-4* locus in *A. franciscana*. E1 to E6 stand for six exons on the DNA scaffold containing the *Af.dsx-4* locus. The red bar indicates the location of the DM domain. The black bar is the open reading frame of *Af.dsx-4,* starting at ATG and stopping at TAG or TAA. The dsx-4αA (Af.dsx^F^) is female-specific splicing, while the dsx-4βC (Af.dsx^M^) is male-specific splicing. The dsx-4αB (Af.dsx^4^) is present in males and females.

The nucleotide sequence BLAST results of isolated Dsx/Dmrt transcripts against the *A. franciscana* genome assembly (NCBI Bioproject PRJNA589114) confirmed these stranscripts are transcribed from different loci on six diferent scaffolds. These loci encoded putatively different DSX proteins bearing one DM domain [28,29]. Among those, the DSX protein that is translated by dsx-2 transcript beared an additional ubiquitin—associated domain (UBA domain) at its C-terminal, which is a common domain found in DSX proteins in insect species [28]. Genome mapping of the full-length of three transcripts from putative dsx-4 contig to the genome of *A. franciscana* revealed a DNA scaffold (scaffold901_size138921) bearing an intact locus, namely Af.dsx-4 locus, which contained six different exons and its arrangement on this scaffold. The organization of six exons on Af.dsx-4 locus and the sequence of dsx-4αB, dsx-4αA, and dsx-4βC transcripts suggest that Af.dsx-4 locus could produces three alternative transcripts including dsx-4αB, dsx-4αA, and dsx-4βC as namely Af.dsx4, Af.dsx^F^ and Af.dsx^M^, respectively. Therefore, these three transcripts of Af.dsx-4 locus are a probable consequence of a splicing event (Figure 2).

**Figure 3:**
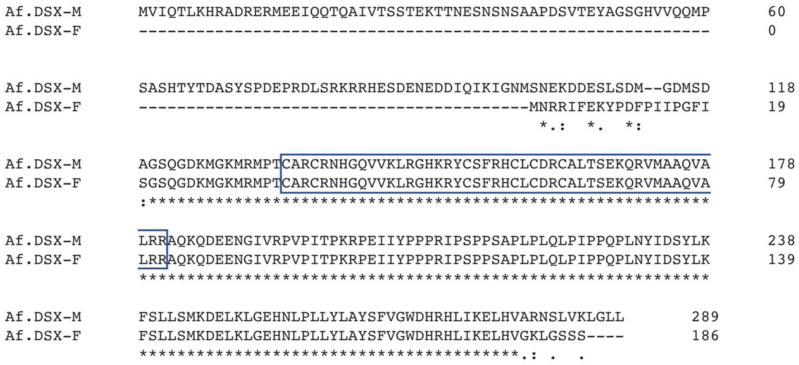
The alignment of primary polypeptide *A. franciscana* Af.DSX-F and Af.DSX-M. The box indicates the DM domain sequence; the asterisks indicate the conserved amino acids.

The open reading frame prediction of Af.dsx^F^ and Af.dsx^4^ transcripts showed that they only contained different 3′UTR sequences which are namely 3′UTR-A and 3′UTR-B, respectively. Therefore, they are predicted to encode for an identical putative polypeptide of 186 aa long, namely Af.DSX-F. In contrast, the Af.dsx^M^ transcript is predicted to encode a polypeptide of 289 aa long, namely Af.DSX-M. The sequence at the 5′end (consisting of a 5′ untranslated region (5′UTR) and a partial CDS encoding for the N-terminal) of Af.dsx^M^ and Af.dsx^F^ transcripts are completely different sequences. Additionally, the Af.dsx^M^ transcript had a different 3′UTR sequence, namely 3′UTR-C, in comparison to the 3′UTR-A and 3′UTR-B sequence of Af.dsx^F^ and Af.dsx^4^ transcripts, respectively. The Af.dsx^4^, Af.dsx^F^ and Af.dsx^M^ transcripts shared a sequence of 477 nucleotides encoding a polypeptide, including the DM domain. The polypeptide alignment suggested that Af.DSX-M is probably an isoform of Af.DSX-F but bears an extended polypeptide at its N-terminal (Figure 3).

### 3.3. Phylogenetic Tree

**Figure 4:**
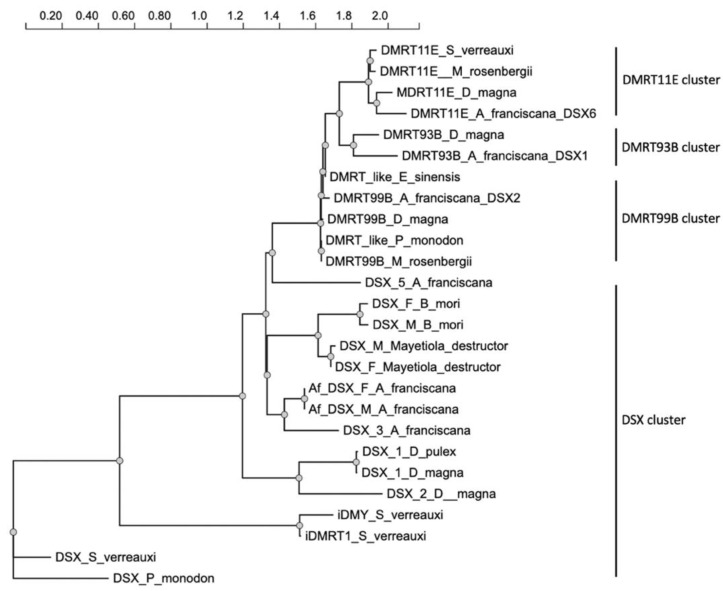
The DSX/DMRT protein phylogenetic tree of *Artemia franciscana* and other arthropods. The phylogeny tree was generated using NGPhylogeny (a free web service) [26].

Eight transcripts were identified encoding seven putative DSX proteins in *A. franciscana*. The polypeptide phylogenetic tree showed that *A. franciscana* DSX-1, DSX-2 and DSX-6 are related to DMRT93B, DMRT99B and DMRT11E protein from crustaceans *D. magna*, *E. sinensis*, *M. rosenbergii* and *S. verreauxi*. In contrast, putative *A. franciscana* DSX-3, Af.DSX-F, Af.DSX-F and DSX-5 polypeptides are closely related to the sex-specific DSX proteins cluster (Figure 4).

### 3.4. Sex-Specific Splicing of Doublesex Gene

**Figure 5:**
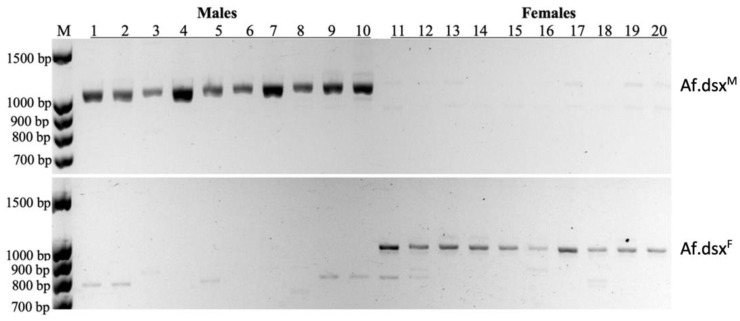
The presence of sex-specific transcript encoded by *Af.dsx-*4 locus in *A. franciscana*. The sex-specific transcripts of Af.dsx^F^ and Af.dsx^F^ as determined by PCR from the cDNA samples of 20 juvenile animals individually. The amplification PCR products of Af.dsx^M^ transcript could only be seen in the male individuals (lanes 1 to 10), while Af.dsx^F^ transcript could only be found in female individuals (lanes 11 to 20). M: DNA marker.

The presence of sex-specific transcripts originating from Af.dsx-4 locus was searched on *A. franciscana* individuals, which were reared separately from the juvenile until the adult stage (full maturity). The sex-specific PCR analysis of Af.dsx^F^ and Af.dsx^F^ transcripts at the juvenile stage was performed on twenty juvenile individuals separately. It again confirmed that Af.dsx^F^ and Af.dsx^M^ were female and male-specific transcripts, respectively (Figure 5).

## 4. Discussion

Extensive studies of the sex determination mechanism in invertebrate animal models such as *D. melanogaster* have shown that sex is determined in the embryonic stage by the involvement of so-called sex-determining genes. Particularly, dsx genes have been identified in many insects to be linked to the sex determination pathway [13]. In *A. franciscana*, the available RNAseq databases suggest that this species has many genes that are similar to sex-determining genes in fruit fly [30]. In D. magna, the transcription of *dmrt11E* and *dmrt99B* is higher in ovaries than in the testes [31]. In *M. rosenbergii*, the transcription of both *dmrt11E* and *dmrt99B* is found in spermatogonia and spermatozoa during spermatogenesis [15]. In *D. magna*, the *dmrt93B* is detected only in the testes, and not in the ovaries [31]. Based on polypeptide phylogenetic analysis, *A. franciscana* DSX-1, DSX-2 and DSX-6 are very close to DMRT93B, DMRT99B and DMRT11E, respectively, thus they are hypothesized to play a similar role in development of gonadal tissue in *Artemia*.

In insects, sex-specific splicing generates sex-specific transcripts from the sex-specific dsx gene controlling sexually-dimorphic traits. The disruption of dsx gene function produces either male-specific sexually-dimorphic defects or intersexual phenotypes [10]. Sex-specific dsx splicing in *B. mori* depends on the presence of MASC protein in the embryo to promote the production of the male-specific splicing variants [32]. Recently, a masc gene has been characterized in *A. franciscana*. Masc RNAi during embryonic development of sexual *A. franciscana* could slightly change the ratio of females/males [9]. This suggests that *A. franciscana* probably also has a similar sex determining mechanism in which the splicing of dsx plays an important role in sex determination. In this study, the RACE method has not been applied on *A. franciscana dsx-3* and *dsx-5*, thus potential variant transcripts of these two genes remain unknown. However, three diferent transcripts of Af.dsx-4 locus were isolated in *A. franciscana* suggesting that a specific splicing mechanism of dsx may exist in *A. franciscana*. In crustaceans, up to now, evidence for alternative splicing of the dsx gene could only be found in *D. magna*, involved in environmental sex determination. Doublesex-1 of *D. magna* is identified to directly determine the gender via the splicing at 5′end of mRNA to produce doublesex-1α or doublesex-1β transcript. Therefore, they have different 5′UTR sequences in their structural transcript, which leads to the control of the translation of key regulator protein of the male phenotype [22,33]. In *A. franciscana*, the Af.dsxM isoform has a different 5′UTR sequence in comparison to the Af.dsx^F^ and Af.dsx^4^ isoforms, thus this may influence the efficient translation to corresponding protein. In comparison to the AF.DSX-F primary polypeptide sequence, AF.DSX-M has a longer N-terminal sequence, this difference could affect the formation of the dimer structure that is needed for its function [34].

Moreover, the transcription of Af.dsx^M^ could only be found in male animals, and Af.dsx^F^ was only present in female animals (Figure 5). This reinforces the hypothesis that sex-specific transcripts of Af.dsx-4 are related to sex determination in *A. franciscana*. Interestingly, Af.dsx^4^ was found present in both sexes (Figure 1B) and since Af.dsx^F^ and Af.dsx^4^ probably encode for the same Af.DSX-F protein, females are predicted to have only Af.DSX-F homodimer. In contrast, the male has both Af.dsx^M^ and Af.dsx^4^, therefore their Af.DSX-F and Af.DSX-M proteins probably are present together, resulting in three different types of protein dimer (Af.DSX-F homodimer, Af.DSX-M homodimer and Af.DSX-F/Af.DSX-M heterodimer). In *B. mori*, the complication of cis- and trans-spliced transcripts of dsx has also been reported to generate seventeen alternatively spliced forms and eleven putative trans-spliced variants. However, all transcripts encode for only four female-specific, two male-specific DSX proteins and one DSX protein common to males and females [35,36]. Therefore, the alternative splicing of Af.dsx-4 locus may need to be investigated further to unravel its precise role in sex determination.

## 5. Conclusions

Six Dsx/*Dmrt* gene family loci were identified on different scaffolds, and their transcripts were sequenced from a cDNA sample of *A. franciscana* in this study. The expression profiles showed that these genes are all expressed in both sexes except the Af.*dsx-4* locus. We determined that the Af.dsx-4 locus potentially transcribes three different isoforms, including *Af.dsx^M^*, *Af.dsx^F^* and *Af.dsx^4^*, probably due to a splicing event. Af.dsx^F^ and Af.dsx^4^ transcripts are found only in female animals, whereas Af.dsx^M^ and Af.dsx^4^ transcripts are detected in male animals. These three transcripts of Af.dsx-4 locus putatively encode Af.DSX-F proteins and Af.DSX-M protein, which is characterized by large differences in amino acid sequence at the N-terminal and minor changes at the C-terminal. Herein, *Af.dsx-4* is a suitable candidate for further investigations into sex determination in *A. franciscana.*

## Figures and Tables

**Table 1:** *PCR* primers used to confirm the *Dsx/Dmrt* transcripts of *A. franciscana*.

Genes	Gene-Specific Primers (5′-3′)	Size in bp
Doublesex-1 (Dmrt93B)	For: ATCCTTTGGGACAATGGATGTTACACTC Rev: CTTTCATAGTGTTGTCAAAACGCGC	903
Doublesex-2 (Dmrt99B)	For: TGGGAGAGAGACAGGAATGTAAATACATAC Rev: GCAGCTCTGAGTGATACCAAAAAATTAAG	1459
Doublesex-3	For: ATATGAAGTGAGCAATGGTGATACGC Rev CGAATTCGTTTAATCACACATTTAAGGCCAC	1169
Af.dsx-4	Af.dsx^F^-For: TCTTCAACCATGAAAACAAACAGGCATCTACC Af.dsx^F^-Rev-A: ATGTTTTCTGGTGTCTTCTCTGTCACAAC	920
	Af.dsx^4^-For: TCTTCAACCATGAAAACAAACAGGCATCTACC Af.dsx^4^-Rev-B: AGAAAGAGATGGTGGAAATTGC	1010
	Af.dsx^M^-For: AGTTCGGTTTGTGGTTCCTCACGG Af.dsx^M^-Rev-C: CAGGCCAATTTTTTGAATATTATCTAGAAGC	1124
Doublesex-5	For: CTACTATCTTACACCTAAGTGCGCCTGAG Rev: GCAACGATTGAAGAGAAATGGGAAAGGATC	918
Doublesex-6 (Dmrt11E)	For: GATTAAACGAACTAAGGCCAGAGAGA Rev: CACAAAGAACGAAAATAAGAAACTAACACAC	966
Tubulin	For: GCAGTGGTCTACAAGGTTTC	605
	Rev: TGCATTGACGTCTTTTGGTACGACATCTC	

## Data Availability

Not applicable.

## References

[1] De Vos S., Bossier P., Van Stappen G., Vercauteren I., Sorgeloos P., Vuylsteke M. (2013). A First AFLP-Based Genetic Linkage Map for Brine Shrimp *Artemia franciscana* and Its Application in Mapping the Sex Locus. PLoS ONE.

[2] Bowen S.T. (1965). The Genetics of *Artemia salina*. V. Crossing over between the X and Y Chromosomes. Genetics.

[3] Parnes S., Khalaila I., Hulata G., Sagi A. (2003). Sex Determination in Crayfish: Are Intersex *Cherax quadricarinatus* (Decapoda, Parastacidae) Genetically Females?. Genet. Res..

[4] Staelens J., Rombaut D., Vercauteren I., Argue B., Benzie J., Vuylsteke M. (2008). High-Density Linkage Maps and Sex-Linked Markers for the Black Tiger Shrimp (*Penaeus monodon*). Genetics.

[5] Ventura T., Sagi A. (2012). The Insulin-like Androgenic Gland Hormone in Crustaceans: From a Single Gene Silencing to a Wide Array of Sexual Manipulation-Based Biotechnologies. Biotechnol. Adv..

[6] Cui Z., Hui M., Liu Y., Song C., Li X., Li Y., Liu L., Shi G., Wang S., Li F. (2015). High-Density Linkage Mapping Aided by Transcriptomics Documents ZW Sex Determination System in the Chinese Mitten Crab *Eriocheir sinensis*. Heredity.

[7] Huylmans A.K., Toups M.A., MacOn A., Gammerdinger W.J., Vicoso B. (2019). Sex-Biased Gene Expression and Dosage Compensation on the Artemia Franciscana Z-Chromosome. Genome Biol Evol.

[8] Jo E., Lee S.J., Choi E., Kim J., Lee J.H., Park H. (2021). Sex-Biased Gene Expression and Isoform Profile of Brine Shrimp Artemia Franciscana by Transcriptome Analysis. Animals.

[9] Li D.-R., Ye H.-L., Yang J.-S., Yang F., Wang M.-R., de Vos S., Vuylsteke M., Sorgeloos P., van Stappen G., Bossier P. (2017). Identification and Characterization of a Masculinizer (Masc) Gene Involved in Sex Differentiation in Artemia. Gene.

[10] Xu J., Zhan S., Chen S., Zeng B., Li Z., James A.A., Tan A., Huang Y. (2017). Sexually Dimorphic Traits in the Silkworm, Bombyx Mori, Are Regulated by Doublesex. Insect Biochem Mol. Biol.

[11] Mawaribuchi S., Ito Y., Ito M. (2019). Independent Evolution for Sex Determination and Differentiation in the DMRT Family in Animals. Biol. Open.

[12] Burtis K.C., Baker B.S. (1989). Drosophila Doublesex Gene Controls Somatic Sexual Differentiation by Producing Alterna-tively Spliced MRNAs Encoding Related Sex-Specific Polypeptides. Cell.

[13] Verhulst E.C., van de Zande L. (2015). Double Nexus—Doublesex Is the Connecting Element in Sex Determination. Brief. Funct. Genom..

[14] Jung H., Yoon B.-H., Kim W.-J., Kim D.-W., Hurwood D.A., Lyons R.E., Salin K.R., Kim H.-S., Baek I., Chand V. (2016). Optimizing Hybrid de Novo Transcriptome Assembly and Extending Genomic Resources for Giant Freshwater Prawns (Macrobrachium Rosenbergii): The Identification of Genes and Markers Associated with Re-production. Int. J. Mol. Sci..

[15] Yu Y.-Q., Ma W.-M., Zeng Q.-G., Qian Y.-Q., Yang J.-S., Yang W.-J. (2014). Molecular Cloning and Sexually Di-morphic Expression of Two Dmrt Genes in the Giant Freshwater Prawn, Macrobrachium Rosenbergii. Agric. Res..

[16] Amterat Abu Abayed F., Manor R., Aflalo E.D., Sagi A. (2019). Screening for Dmrt Genes from Embryo to Mature Macro-brachium Rosenbergii Prawns. Gen. Comp. Endocrinol..

[17] Zhong P., Zhou T., Zhang Y., Chen Y., Yi J., Lin W., Guo Z., Xu A., Yang S., Chan S. (2019). Potential Involvement of a DMRT Family Member (Mr-Dsx) in the Regulation of Sexual Differentiation and Moulting in the Giant River Prawn Macrobrachium Rosenbergii. Aquac. Res..

[18] Ma K., Qiu G., Feng J., Li J. (2012). Transcriptome Analysis of the Oriental River Prawn, Macrobrachium Nipponense Using 454 Pyrosequencing for Discovery of Genes and Markers. PLoS ONE.

[19] Zhang E.F., Qiu G.F. (2010). A Novel Dmrt Gene Is Specifically Expressed in the Testis of Chinese Mitten Crab, Eriocheir Sinensis. Dev. Genes. Evol..

[20] Li S., Li F., Yu K., Xiang J. (2018). Identification and Characterization of a Doublesex Gene Which Regulates the Expression of Insulin-like Androgenic Gland Hormone in Fenneropenaeus Chinensis. Gene.

[21] Peng J., Wei P., Zhang B., Zhao Y., Zeng D., Chen X., Li M., Chen X. (2015). Gonadal Transcriptomic Analysis and Dif-ferentially Expressed Genes in the Testis and Ovary of the Pacific White Shrimp (Litopenaeus Vannamei). BMC Genom..

[22] Kato Y., Kobayashi K., Watanabe H., Iguchi T. (2011). Environmental Sex Determination in the Branchiopod Crustacean Daphnia Magna: Deep Consevation of a Doublesex Gene in the Sex-Determining Pathway. PLoS Genet..

[23] Chandler J.C., Fitzgibbon Q.P., Smith G., Elizur A., Ventura T. (2017). Y-Linked IDmrt1 Paralogue (IDMY) in the Eastern Spiny Lobster, Sagmariasus Verreauxi: The First Invertebrate Sex-Linked Dmrt. Dev. Biol..

[24] De Vos S., van Stappen G., Sorgeloos P., Vuylsteke M., Rombauts S., Bossier P. (2019). Identification of Salt Stress Response Genes Using the Artemia Transcriptome. Aquaculture.

[25] Altschul S.F., Madden T.L., Schäffer A.A., Zhang J., Zhang Z. (1997). Gapped BLAST and PSI-BLAST: A New Generation of Protein Database Search Programs. Nucleic Acids Res..

[26] Lemoine F., Correia D., Lefort V., Doppelt-Azeroual O., Mareuil F., Cohen-Boulakia S., Gascuel O. (2019). NGPhyloge-ny.Fr: New Generation Phylogenetic Services for Non-Specialists. Nucleic Acids Res..

[27] Yi W., Zarkower D. (1999). Similarity of DNA Binding and Transcriptional Regulation by Caenorhabditis Elegans MAB-3 and Drosophila Melanogaster DSX Suggests Conservation of Sex Determining Mechanisms. Development.

[28] Bayrer J.R., Zhang W., Weiss M.A. (2005). Dimerization of Doublesex Is Mediated by a Cryptic Ubiquitin-Associated Domain Fold: Implications for Sex-Specific Gene Regulation. J. Biol. Chem..

[29] An W., Cho S., Ishii H., Wensink P.C. (1996). Sex-Specific and Non-Sex-Specific Oligomerization Domains in Both of the Doublesex Transcription Factors from Drosophila Melanogaster. Mol. Cell. Biol..

[30] De Vos S. (2014). Genomic Tools and Sex Determination in the Extremophile Brine Shrimp *Artemia franciscana*. Ph.D. Thesis.

[31] Kato Y., Kobayashi K., Oda S., Colbourn J.K., Tatarazako N., Watanabe H., Iguchi T. (2008). Molecular Cloning and Sexually Dimorphic Expression of DM-Domain Genes in Daphnia Magna. Genomics.

[32] Kiuchi T., Koga H., Kawamoto M., Shoji K., Sakai H., Arai Y. (2014). A Single Female-Specific PiRNA Is the Primary Determiner of Sex in the Silkworm. Nature.

[33] Nong Q.D., Matsuura T., Kato Y., Watanabe H. (2020). Two Doublesex1 Mutants Revealed a Tunable Gene Network Underlying Intersexuality in *Daphnia magna*. PLoS ONE.

[34] Zhang W., Li B., Singh R., Narendra U., Zhu L., Weiss M.A. (2006). Regulation of Sexual Dimorphism: Mutational and Chemogenetic Analysis of the Doublesex DM Domain. Mol. Cell. Biol..

[35] Duan J., Xu H., Guo H., O’Brochta D.A., Wang F., Ma S., Zhang L., Zha X., Zhao P., Xia Q. (2013). New Insights into the Genomic Organization and Splicing of the Doublesex Gene, a Terminal Regulator of Sexual Differ-entiation in the Silkworm Bombyx Mori. PLoS ONE.

[36] Duan J., Xu H., Wang F., Ma S., Zha X., Guo H., Zhao P., Xia Q. (2013). Novel Female-Specific Trans-Spliced and Al-ternative Splice Forms of Dsx in the Silkworm Bombyx Mori. Biochem. Biophys. Res. Commun..

